# Evaluation of the Metabolic Relationship between Cows and Calves by Monitoring Calf Health and Cow Automatic Milking System and Metabolic Parameters

**DOI:** 10.3390/ani13162576

**Published:** 2023-08-10

**Authors:** Mingaudas Urbutis, Dovilė Malašauskienė, Mindaugas Televičius, Vida Juozaitienė, Walter Baumgartner, Ramūnas Antanaitis

**Affiliations:** 1 Large Animal Clinic, Veterinary Academy, Lithuanian University of Health Sciences, LT-47181 Kaunas, Lithuania; dovile.malasauskiene@lsmuni.lt (D.M.); mindaugas.televicius@lsmuni.lt (M.T.); ramunas.antanaitis@lsmuni.lt (R.A.); 2 Department of Biology, Faculty of Natural Sciences, Vytautas Magnus University, LT-44248 Kaunas, Lithuania; 3 University Clinic for Ruminants, University of Veterinary Medicine, A-1210 Vienna, Austria

**Keywords:** calf metabolism, herd management, cow metabolism, biomarkers, negative energy balance, calf health monitoring

## Abstract

**Simple Summary:**

The relationship of a pregnant cow and the calf in her womb has yet to be studied thoroughly. There are signs that the metabolic state of a cow, such as obesity or malnutrition, during pregnancy can have an effect on the health status not only of the cow, but of her calf as well. We wanted to check whether there was a relationship and decided to examine 40 cows and their calves for 30 days after calving. The results indicated that mothers of calves who were more prone to disease and showed more symptoms were also more at risk of developing metabolic diseases during the 30-day period—indicated by higher milk yield, greater milk fat concentration and higher milk fat to protein ratio together with lower milk lactose concentration. This study shows signs that health status might be predetermined during pregnancy and drives us to further investigate this more thoroughly. In doing so, farmers and veterinary health practitioners might have a greater impact on the pregnancy period of a cow and guarantee a healthy life for her and her offspring.

**Abstract:**

With this study, we investigated the relationship between a cow’s and calf’s metabolic state, and its effect on health status. To achieve this, 20 calves of primiparous and 20 calves of multiparous cows were selected. The calves were monitored for 30 days and scored for signs of disease, as described in McQuirk (2008); according to score, they were divided into healthier calves; the Low calf score group (LCS, 5–8), Medium calf score group (MCS, 9–12) and High calf score group (HCS, 14–17); or calves most prone to disease. Their mothers were monitored for the same period with a Lely Astronaut 3 herd management system (Lely, Maassluis, The Netherlands) for rumination time, milk yield, milk fat, protein, lactose concentrations and milk fat to protein ratio. Both cows and calves were sampled for blood, and concentrations of glucose with β-hydroxybutyrate were registered. The results indicate that primiparous cows had a 16% higher blood glucose concentration (3.03 mmol/L SE = 0.093) compared with multiparous cows (2.61 mmol/L, SE = 0.102) (*p* < 0.01), but no difference in calf glucose was recorded. Β-hydroxybutyrate levels did not differ significantly between cows and calves by parity group. Rumination time was longest in the HCS group at 550.79 min/d. and was 16% longer compared with the LCS group (461.94 min/d.; *p* < 0.001) and 8% longer compared with the MCS group (505.56 min/d.; *p* < 0.001). The MCS group rumination time mean was statistically significantly higher compared with the LCS group by 8% (*p* < 0.001). Milk yield was also highest in the HCS group (44.8 kg/d.): 19% higher compared with the MCS group (36.31 kg/d., *p* < 0.001) and 13% higher than the LCS group (38.83 kg/d., *p* < 0.001). There was also a significant difference between the MCS and LCS groups of 6% (*p* < 0.001). The HCS group had the highest milk fat concentration (4.47%): it was 4% higher compared with the LCS group (4.28%, *p* < 0.001) and 5% higher than the MCS group (4.25%, *p* < 0.001). Milk fat to protein ratio was highest in the HCS group (1.21) and was 7% higher than in the MCS group (1.12, *p* < 0.001) and 8% higher than in the LCS group (1.11, *p* < 0.001). The LCS group was determined to have the highest concentration of milk lactose (4.66%). It was 1% higher compared with the MCS group (4.62%, *p* < 0.001) and 1.07% higher than the HCS group (4.61%, *p* < 0.001). We can conclude that parity did not affect calf health status and that cows of the HCS group showed symptoms of negative energy balance expressed through higher milk yield, higher milk fat concentration and higher milk fat to protein ratio, with lower milk lactose concentration. Further and more thorough research is needed to evaluate the relationship between pregnant cows and calves.

## 1. Introduction

Recent research points to the fact that the metabolic status of a cow before parturition can have an effect on the immunoglobulin transfer to its calf and affect its health status [1]. The transition period is associated with negative energy balance, which in turn causes lipomobilization and ketogenesis [2]. The main blood indicators of lipid mobilization in dairy cows are β-hydroxybutyrate (BHB) and non-esterified fatty acids (NEFAs) [3]. Increased NEFA concentrations, often found in the blood of obese cows, are a sign of a higher risk of disease in transition cows [4]. Changes in BHB and milk fat concentration levels indicate the synthesis of ketone bodies resulting from lipomobilization [5,6].

The metabolites found in a cow’s circulation during this period influence the compositions of colostrum and newborn calf blood serum. In one study, it was found that circulating NEFA concentrations were connected to lipids in circulation at 5 weeks and 1 week before parturition, as well as to the lipids found in colostrum. The variety and concentrations of lipid and membrane tri-acyl-glycerols (TGs) were alike in both cow plasma and calf serum [7]. During the first days of lactation, the main source of milk fat in the mammary cells is used-up NEFAs; therefore, with elevated plasma NEFA concentrations, variety in the fatty acid composition in milk is usually seen [8]. A negative relationship was registered between the NEFA area under the curve (AUC) and the membrane lipid concentration and total phosphatidylglycerol (PG) in first milk. At the same time, the NEFA AUC had a positive correlation with the total TG content in plasma at one week prior to parturition. These results point toward the conclusion that, at 7 days before parturition, the registered higher TG lipid concentration was influenced by increasing NEFA concentrations, but it negatively affected the membrane lipid and PG concentrations in [7]. Some studies add that this increase in the TG-to-membrane lipid ratio could affect milk fat globule (MFG) synthesis, making it significantly larger [9].

The concentration of phosphatidylglycerol (PG) was higher in calf serum and colostrum in comparison with cow plasma; from the 24 PGs found in colostrum, 23 of them were also found in calf serum. With increasing NEFA concentrations and the changing metabolic status of the dam, the circulation of lipids and colostrum lipid concentrations tends to change as well. Despite the similar lipidomes of cows and calves, the colostrum lipidome status is different, apart from the PG concentration and variety. Colostrum may be the main source of PGs found in calves [7].

Negative energy balance in a cow and fat mobilization modifies the colostrum contents, in turn affecting a calf’s immune response. In one study, it was determined that higher concentrations of NEFAs were negatively correlated with the phosphatidylglycerol (PG) concentrations of colostrum. PG is one of the most abundant phospholipids in colostrum [7]. It is necessary for the immune system [10,11] and cell mitochondria [12].

Data from another study indicate that lactation number may also have an influence on metabolic status and the blood serum and colostrum contents. In the aforementioned study, the differences in TG and FA composition observed between the multiparous (M) and primiparous (P) cow colostrum samples suggested that these groups of cows had a different response to higher energy demands due to the rapidly increasing milk yield. Taking possible individual variations in its contents into consideration, the first colostrum is still enriched with essential molecules—cholesterol, n-3 FA and plasma lipids (PLs) (sphingomyelin in particular) [13]. Cholesterol is one of the main structural components of cell membranes [14]. Moreover, it is the precursor for steroid hormones and plays a major role in the development and pattern formation of the central nervous system [15]. A high concentration of PLs, mostly sphingomyelin, during the first hours of life illustrates the importance of these compounds—they form the membrane structure, act as transducers of biological signals and might enhance the protection of the intestinal tract against infection [16]. Thus, it is evident that a proper metabolic profile of the pregnant cow should be maintained to provide the best quality colostrum for its newborn calf.

Early diagnosis of metabolic diseases in prepartum cows is fairly difficult, since the majority of novel diagnostic tools and systems are based on monitoring changes in milk components [17]. To evaluate the metabolic status in the prepartum period, researchers must rely on blood sampling [18]. However, the prepartum period has a significant effect on the early lactation period, which can be closely monitored via automatic milking systems and used to diagnose metabolic and other diseases [17,19]. Often, subclinical ketosis-positive milk samples are associated with an elevated somatic cell count (SCC)—significantly higher SCC values were observed in BHB samples ≥ 0.10 mmol/L [20,21]. In one study, BHB-positive samples had significantly higher means for milk fat proportion and SCC, while a significantly lower mean for milk protein proportion was observed. Considering the parity of animals, milk fat, milk protein and milk lactose production (kg/d.) were all greater in multiparous than in primiparous cows in another study. However, the concentration of NEFAs and BHB were not different between parities. The proportion of cows with subclinical ketosis was 68.6% (24/35) for primiparous cows and 57.9% (33/57) for multiparous cows, and there was no relation with parity [22,23].

Seeing evidence of the cows’ metabolism affecting the colostrum quality and, in turn, the calves, we hypothesized that cows with predetermined metabolic diseases, registered by fluctuations in automatic milking system parameters, would give birth to calves that would also be more susceptible to disease. The aim of the study was to monitor the metabolic status of cows by using the parameters registered by an automatic milking system and at the same time to monitor the health status of their calves. The investigation would give more insight into the relationship between the metabolism of cows and calves.

## 2. Materials and Methods

### 2.1. Ethical Approval

During the study, all of the procedures followed the Lithuanian Law on Animal Welfare and Protection. The trial was approved under the number G2-227 after a thorough review of the methods by the State Food and Veterinary Service’s Department of Animal Welfare.

### 2.2. Animals’ Farm and Feeding

The study was carried out on a dairy farm situated in the middle part of Lithuania at 54°58′34.9″ N 23°46′04.2″ E coordinates. The farm had around 1200 milking cows. It was a free-stall barn with robotic milking performed by Lely Astronaut 3 milking robots (Lely, Maassluis, The Netherlands). The cows were fed a total mixed ration and both primiparous and multiparous received the same feed, balanced to fit their physiological and production needs for a 550 kg Holstein-Friesian cow producing 40 kg of milk per day on average [24]. Total mixed rations consisted of 24% corn silage, 5% grass hay, 16% grass silage, 50% grain concentrate slurry and 5% mineral mixture. Dry matter made up 48.00% or rations, of which 20% was acid detergent fiber, 39% non-fiber carbohydrates, 28% neutral detergent fiber and 16% crude protein. Cows were fed at 5 A.M. and 5 P.M. daily all year round. Each cow weighed 550 ± 25 kg on average. During 2022, the milk production was around 12,000 kg per cow per year.

In total, 20 calves from primiparous cows (2 years of age) and 20 calves from multiparous cows (only 2nd and 3rd lactation, 3 and 4 years, respectively) were chosen for this study. The calves were born during the period between 25 May 2022 and 25 April 2022. Since calving happened during the same season in a period of one month, the seasonal and climatic effect on the animals was similar for all participants. For the calves and cows to be included in the study, the calving had to be easy (no assistance from veterinary professionals required), cow was healthy prior to the calving and the calf received good quality colostrum from its mother. The calves were given 4 L of colostrum from their dam in the first hour after birth by trained farm staff members [25]. Before being fed colostrum, it was tested with MS Colostro Balls (MS Schippers, Kerken, Germany)—specially made colorful tablets that each have a different density and an appropriate color. The test evaluates the density of the first colostrum—if all of the tablets float, this indicates that the density of the colostrum is around 1075 g/dm^3^. The test was performed at the required temperature of 20–30 °C, according to the manual. All of the colostrum provided for the calves had a density of around 1075 g/dm^3^, indicated as “very good” on the chart provided with the product [26]. After 12 h, a second dose of colostrum was provided for the calves. Calves were then separated into single pens and started on milk replacer (Sprayfo Yellow, Trouwnutrition, Putten, The Netherlands) and concentrated feed. Milk replacer was composed of whey powder, 10% skimmed milk powder, coconut and palm oil and hydrolyzed wheat proteins with vitamins and minerals. The composition was as follows: 21.5% crude protein, 17.5% crude oils and fats, 9% crude ash and 0% crude fiber. In addition, each kilogram of milk replacer was enriched with 25,000 IU Vitamin A, 5.000 IU Vitamin D3, 300 mg of Vitamin E, 0.3 mg of selenium, 10 mg of copper and 90 mg of iron. The solution was mixed with water at 45–55 °C at a ratio of 130–140 g per liter. A total of 4 L of replacer was fed to the calves twice a day every 12 h. The temperature at time of feeding was 40 °C.

### 2.3. Research Design

On the 3rd day of life, a clinical examination was performed for the calves (*n* = 42, one of the cows had a twin birth; therefore, the number of calves is higher compared with that of cows) by a trained veterinary professional with 5 years of work experience. The methodology for health evaluation was based on McQuirck (2008)—calves were evaluated for nasal discharge, eye discharge, ear position, rectal temperature, diarrhea and presence of disease [27]. In this scheme, a score of 0 represents a lack of clinical symptoms and indicates a healthy calf, while presence of other symptoms and their severity indicate a possible disease. We did not record clinical diagnoses such as pneumonia and/or diarrhea, as the scoring system is not designed that way—a higher score in multiple examined symptoms is a good indicator of disease. Calves included in the study were clinically examined every 2–3 days for a period of 3 weeks. After three weeks, a total score for each calf was calculated by summing up the scores of each clinical examination. After analyzing scores, a class interval of “4” was used to assign calves into distinct groups. Calves with a score of 5–8 were assigned to the Low calf score group (LCS)—calves that showed the least symptoms (*n* = 19). Since no calf had a score of “13”, the Medium calf score group (MCS) consisted of calves that reached a score of 9–12 (*n* = 16). The High calf score group (HCS) consisted of calves that scored 14–17 points (*n* = 7).

### 2.4. Measurements

A blood sample via jugular venipuncture was taken from calves on the 3rd day of life into a blood biochemistry tube (BD Vacutainer Red, Mississauga, ON, Canada) without any conservatives. A drop of blood was drawn from the tube and used in a hand-held blood glucose (Glu) and β-hydroxybutyrate (BHB) meter (CentriVet GK, Acon, San Diego, CA, USA). The blood samples were transported in 4 °C to the laboratory for further analysis within an hour from sampling. In the laboratory, the samples were centrifuged at 1200 RPM for 8 min. The centrifuged serum was then analyzed to determine the serum protein concentration and to evaluate the presence of failed passive transfer. The sample serum was evaluated with a hand-held refractometer (RHC200, YHequipment, Shenzhen, China). According to Renaud et al., (2018), the threshold for failed passive transfer is <5.2 g/dL [28]. All calves had an average serum protein concentration of 7.6 g/dL—all calves had adequate passive immunity.

On the same day the calves were clinically examined and sampled, a blood sample was also taken from their dam. A blood sample from the coccygeal vein was taken into a red biochemistry tube (BD Vacutainer, Mississauga, ON, Canada); a drop of blood was used to determine the concentration of Glu and BHB by using a hand-held device (CentriVet GK, Acon, San Diego, CA, USA). The samplings for both cows and calves were performed once on the third day of life and lactation.

After 3 weeks of lactation for each cow, data from automated milking system Lely Astronaut A3 (Lely, Maassluis, The Netherlands) were retrieved and analyzed. Parameters such as rumination time (RT) (duration of rumination in minutes per day), milk yield (MY) (kilograms of milk produced per day), milk protein content (MP) (percentage of milk protein in milk), milk fat content (MF) (percentage of milk fat in milk), milk lactose concentration (ML) (percentage of milk lactose in milk) and milk fat and protein ratio (MF:P) were monitored. These parameters were registered each day for each cow and the mean value of the 3 week period was used for statistical analysis.

#### Statistical Analysis

Shapiro–Wilk test was used to determine the normal distribution of blood biochemistry data, as the number of measurements was low. Cow BHB and Calf BHB data were not normally distributed. For normally distributed data of cow and calf glucose concentrations, a difference between parity groups was determined using One-way ANOVA. The results were presented as means and standard deviation. Correlation analysis of normally distributed parameters was performed via Pearson correlation and the strength of the correlation was set as follows: |0.1–0.3|—low, |0.3–0.5|—moderate and |0.5–1.0|—strong. Non-normally distributed data were analyzed with the Mann–Whitney U test and the distribution of calf score class between parity groups was evaluated using Pearson Chi-Square.

Kolmogorov–Smirnov test was used to evaluate the normal distribution of the milk parameters and data from automatic milking system since measurement count was high. Parameters of rumination time, milk yield, milk protein concentration, milk fat concentration, milk fat and protein ratio and milk lactose concentration were normally distributed. Student’s *t*-test was used to evaluate the difference in means of milking parameters between primiparous and multiparous cows. One-way ANOVA was used to evaluate if there was a significant variation in parameters between cows in groups according to the calf score. LSD (least significant difference) post hoc test was used to determine which groups of calf score differed significantly from each other. The results were presented as means with standard error. Degree of significance was set to *p* < 0.05.

## 3. Results

### 3.1. Blood Biochemistry of Cows and Calves

Glucose concentration was significantly different between parity groups (Table 1). Primiparous cows had a 16% higher blood glucose concentration (3.03 mmol/L, Std. error ± 0.093) compared with multiparous cows (2.61 mmol/L, Std. error ± 0.102) (*p* < 0.01). There was no significant difference in glucose concentration between calves of different parity groups (*p* > 0.05).

No significant difference in BHB concentration between parity groups of cows was recorded (Table 2)—primiparous cows had a mean rank of 18.65 and multiparous cows had a mean rank of 22.35 (U = 163, *n* = 40, *p* > 0.05). Calf BHB was not significantly different between groups as well—the primiparous group had a mean rank of 21.57 and that of the multiparous group was 21.43 (U = 219, *n* = 42, *p* > 0.05).

When evaluating the distribution of calves’ score class between parity groups, no significant difference was also recorded (*χ2*(2) = 1.588, *p* > 0.05) (Table 3). There was no significant difference between the Low calf score class and the Medium (*p* > 0.05) and High score classes (*p* > 0.05), as well as between the Medium and High score classes (*p* > 0.05).

A significant moderate negative correlation was calculated between cow BHB concentration and cow glucose concentration (*r* = −0.353, *p* < 0.05) (Table 4). Between cow BHB and calf BHB concentrations, a significant moderate negative correlation (*r* = −0.476, *p* < 0.01) was also determined.

### 3.2. Herd Health Management and Milking Parameters of Primiparous and Multiparous Cows

Student’s *t*-test revealed significant differences in milking parameters between parity groups (Table 5). Primiparous cows had a 6% lower rumination time compared with multiparous cows (Std. error ± 6.086, *p* < 0.001). Multiparous cows had a significantly larger milk yield compared with primiparous cows—a difference of 32% (Std. error ± 0.49, *p* < 0.001). Milk protein concentration also differed between groups—multiparous cows had 3.81%, while primiparous protein concentration was 3.77% (Std. error ± 0.016, *p* < 0.01). Multiparous cows had a higher milk fat percentage compared with primiparous cows, which amounted to a difference of 3% (Std. error ± 0.032, *p* < 0.001). This can also be seen in the difference in the milk fat to protein ratio—multiparous cows had a ratio of 1.15, whereas primiparous cows had a lower ratio of 1.12—a difference of 3% (Std. error ± 0.008, *p* < 0.001). Milk lactose concentration was also higher in multiparous cows—4.67% compared with 4.61% in primiparous cows. It was higher by 2% (Std. error ± 0.004, *p* < 0.001).

### 3.3. Herd Health Management and Milking Parameters According to Calf Score Groups

There were significant differences in automatic milking system parameter means between calf score groups (Table 6). Rumination times were highest in the High calf score group, followed by the Medium calf score group and the Low calf score group (*F* = 61.86, *p* < 0.001). Milk yield was also the highest in the High calf score group, followed by the Low calf score group and the Medium calf score group (*F* = 52.42, *p* < 0.001). The Low calf score group had the highest milk protein concentration, followed by the Medium calf score group and the High calf score group (*F* = 29.17, *p* < 0.001). Milk fat concentration was lowest in the Medium calf score group, preceded by the Low calf score group, and the highest concentration was found in the High calf score group (*F* = 11.45, *p* < 0.001). The milk fat to protein ratio was also highest in the High calf score group, followed by the Medium calf score group and the Low calf score group (*F* = 47.56, *p* < 0.001). Milk lactose concentration was lowest in the High calf score group. The Medium calf score group had a higher milk lactose concentration, and the highest concentration was in the Low calf score group (*F* = 38.76, *p* < 0.001). Further analysis of differences in means between groups is presented further below.

Post hoc analysis revealed significant differences in milking parameters between calf score groups (Table 7). Rumination times were longest in the HCS group and were 16% longer compared with that of the LCS (*p* < 0.001) and 8% longer compared with the MCS (*p* < 0.001). The MCS rumination time mean was statistically significantly higher compared with that of the LCS as well, by 8% (*p* < 0.001). Milk yield was also highest in the HCS group—19% higher compared with that of the MCS (*p* < 0.001) and 13% higher than the LCS (*p* < 0.001). There was also a significant difference between the MCS and LCS groups—6% (*p* < 0.001). Milk protein concentration was highest in the LCS group—1.5% higher than that in the MCS (*p* < 0.001) and 4% higher than the HCS (*p* < 0.001). The difference between the MCS and LCS was also statistically significant (*p* < 0.001). The HCS group had the highest milk fat concentration—it was 4% higher compared with that of the LCS group (*p* < 0.001) and 5% higher than the MCS group (*p* < 0.001). Though the LCS group had a higher concentration compared with the MCS, this difference was not significant (*p* > 0.05). Milk fat to protein ratio was highest in the HCS group and was 7% higher than in the MCS (*p* < 0.001) and 8% higher than in the LCS (*p* < 0.001). No significant difference between the LCS and MCS groups in this parameter has been calculated—*p* > 0.05. The LCS group was determined to have the highest concentration of milk lactose. It was 1% higher compared with that of the MCS (*p* < 0.001) and 1.07% higher than the HCS group (*p* < 0.001). As with other milk quality parameters, there was no significant difference between the LCS and MCS groups—*p* > 0.05.

From these differences, we can see that cows in the High calf score group have more clear signs of negative energy balance expressed by the highest milk yield, highest milk fat concentration and milk fat to protein ratio, coupled with the lowest milk protein and lactose concentrations.

## 4. Discussion

The primiparous cow group had a higher blood serum glucose concentration compared with the multiparous cow group and a similar BHB concentration. Similar results were registered by van Knegsel et al. [29]. A higher blood glucose concentration has been registered in other studies as well and it has been concluded that primiparous cows, as they have a lower milk yield on average, do not require high amounts of glucose for tissues and metabolism; therefore, higher concentrations of it is detected in the blood [30,31]. Blood BHB concentration did not differ statistically significantly between groups and also agrees with the data from van Knegsel et al.’s study [29]. A lower glucose concentration together with a stable BHB concentration in the multiparous group points to a manageable energy balance, or at least indicates that blood indices have not been affected yet [32].

In this study, multiparous cows showed better performance results compared with primiparous cows. Multiparous cows had a higher milk yield by 32% compared with primiparous cows. A higher milk yield in multiparous cows is in accordance with the work of Wathes et al., where a significant difference (*p* < 0.05) in milk yield was recorded throughout the study period between multiparous and primiparous animals [31]. Similar results were provided by Marumo et al., where multiparous cows had a mean milk yield of 22.7 kg/d compared with 12.7 kg/d of primiparous cows (*p* < 0.05) [33]. A higher milk yield in multiparous cows compared with primiparous cows is natural, since the most intense proliferation of mammary cells is happening during the dry period and the quantity of mammary cells closely correlates with milk yield [34]. A longer rumination time in multiparous cows was also registered in our study. These results agree with the study of Maekawa et al., where multiparous cows spent more time ruminating compared with primiparous cows—560 vs. 508 min/d [35]. One explanation for this is that multiparous cows have a higher dry matter intake and require more saliva and more thorough breakdown of feedstuff for efficient fermentation [36,37]. Milk composition also differed in multiparous cows compared with primiparous cows and pointed to a better performance—milk fat, protein and lactose concentrations were higher. In the work of Colebrander et al. and Maekawa et al., parity had no effect on milk fat percentage [35,38]. A decrease in milk protein and lactose concentration in the multiparous cow group, while milk fat concentration stayed similar, was noted in another study as well [39]. Yet, in another experiment, the same authors modified the feed and obtained different results—multiparous cows produced more milk but with a lower milk fat percentage [40]. Taking this into consideration, other authors have concluded that milk composition is more influenced by lactation stage, feed quality and composition together with fiber quantity [41]. On the other hand, in the study of Gärtner et al., multiparous cows showed a higher milk fat concentration but a similar milk protein concentration compared with primiparous cows. The authors claim that multiparous cows with their increased milk yield have to utilize fat metabolism for energy production, which results in more volatile fatty acids in blood circulation and milk [42].

In our research, we found a moderate negative correlation (*r* = −0.476) between cows’ BHB and calves’ BHB. A physiological decrease in blood BHB concentration in calves was recorded by Dänicke et al. [43]. Similar results concerning the decrease in calf BHB serum concentration in the first days was also recorded in the study of Collazos et al. [44]. Accompanying the decrease in BHB concentration was also a lower concentration of NEFAs. On the other hand, glucose concentration showed a tendency to increase. Lower concentrations of calf BHB can be linked to adequate and elevated nutrition of good quality colostrum and milk replacer, providing the calf with sufficient energy [45]. Concerning the negative correlation of metabolic markers in cows and calves, the paper of Immler et al. also states that cows with higher NEFA concentrations had calves with higher serum IgC concentrations [1]. Even though there are data that an NEB of a cow has a positive effect on the metabolism of calves’ and cows’ colostrum quality, other studies indicate that higher maternal NEFA concentrations have a negative effect on the body weight and immune response of calves [46,47]. The results in the literature differ and are explained by a variety of factors that could influence the cows’ and calves’ metabolism; therefore, more studies are needed to determine the reasons for this relationship and how it can be used to benefit both cows and their calves [48,49].

The results indicate that cows showing signs of NEB birthed calves more prone to disease. Cows suffering from negative energy balance or subclinical ketosis tend to prioritize milk production in comparison with their metabolic needs [50,51]. In the study of Ha et al., cows suffering from clinical ketosis produced significantly more milk compared with cows with subclinical ketosis. In the same study, the lowest milk production was recorded by non-ketotic cows. These differences were observed only on the 4th–6th day period and following lactation—cows with clinical ketosis were overtaken by subclinical and non-ketotic cows in milk yield [48]. On the other hand, in the research of Mellado et al., ketotic cows had a higher 305 day milk yield compared with non-ketotic cows [51]. In our study, cows in the HCS group had a higher milk yield compared with those in the LCS group.

Together with a higher milk yield, HCS group cows also had a longer rumination time. High milk production demands a significant amount of nutrients and energy from the cow, which in turn causes a negative energy balance [52]. Propionate, a necessary fatty acid for gluconeogenesis, is fermented in the rumen and is the main glucose precursor for cows [53]. Cows with longer rumination times show a higher rumen protozoa count and activity [54]. In turn, a longer rumination time also translates into a higher milk yield [55]. Cows in the HCS group were at a higher risk of a negative energy balance and metabolic disease, indicated by a higher milk yield supported by a higher rumination time.

A higher risk of negative energy balance for HCS group cows was also indicated by other milk parameters registered by the automatic milking system. HCS group cows had a higher concentration of milk fat, but a lower concentration of milk protein compared with LCS group cows. Naturally, this also resulted in a higher milk fat and protein ratio in the HCS group cows compared with the LCS group cows. The parameters of milk fat, protein and their ratio can be used as a sign of negative energy balance. In the study conducted by Toni et al., cows with a milk fat to protein ratio of 1.5–2 had a higher milk yield, compared with cows with a fat to protein ratio of <1, and had a higher incidence of metabolic and reproductive diseases throughout lactation [56]. The physiology of a cow prioritizes milk production and the use of body fat reserves in a negative energy balance state. This is evident by an increase in milk fat percentage and a drop in milk protein percentage whilst maintaining milk yield [57]. It is also important to note that in the same study by Toni et al., cows with a milk fat to protein ratio of 2–3 had a lower milk yield compared with cows with 1.5–2 [56]. This is in agreement with data of Bellato et al., where a negative correlation between milk yield and milk fat percentage was registered [58]. An increasing milk fat to protein ratio correlates with an increasing blood BHB concentration and is an indication of hyperketonemia and negative energy balance [59,60].

Milk lactose concentration was also lower in the HCS group cows. A low milk lactose percentage indicates a lack of energy reserves. In the study of Televičius et al., the cow group with <4.70% milk lactose concentration had a higher incidence of metabolic diseases evaluated by the fat to protein ratio. Nonetheless, the group with a higher milk lactose concentration showed lower risk of mastitis, indicated by lower milk electrical conductivity, and was 26 times more likely to become pregnant [61]. Furthermore, milk lactose concentration can be used as an indicator for subclinical mastitis, since there is a negative correlation with somatic cell count [62]. Also, lactose concentration in milk is one of the most important factors for a higher milk yield. By being an osmotic regulator, it draws more water from the bloodstream and increases the quantity of milk [63,64]. When, in a negative energy balance state, blood glucose concentration, a primary source for milk lactose, is low, it negatively affects cows’ milk yield and increases the risk of mastitis [65].

The prepartum period can determine the health status of not only the cows’ upcoming lactation but that of her offspring as well. This is evident in the research of Noya et al., where underweight cows in the dry period gave birth to offspring with lower body measurements at weaning time, a decreased average daily gain and lower plasma insulin-like growth factor-1. At the same time, underweight cows showed a lower colostrum immunoglobulin G concentration and a higher milk fat concentration [66]. Supplementation during the dry period can directly affect the performance results and health status of the unborn calf, but more studies are needed to investigate this phenomenon [67]. Our study shows signs of this relationship between cows’ and calves’ metabolism. We must note, however, that this study is not without shortcomings—future studies should include more study subjects per group and a more thorough evaluation of the metabolic and health parameters of both cows and calves should be performed. Doing this will produce better results and the relationships between parameters will become more evident. Additionally, environmental and physiological factors such as heat stress and estrus should be taken into consideration, as they can impact the health status of animals. Monitoring the feeding behavior (rumination, eating, drinking time and chewing motions) during the dry period and early lactation would be beneficial as well, since these are established health parameters that are registered with novel technologies. With more research, chances are that a more significant impact on the health status of cows’ dry period could be achieved. In doing so, the health of both fresh cows and calves might be improved and better results could be reached.

## 5. Conclusions

Multiparous cows had a better performance compared with primiparous cows, described by a significantly higher milk yield, longer rumination duration and higher concentration of milk components. A better performance also meant a bigger risk for negative energy balance—indicated by a higher milk fat to protein ratio in the multiparous cow group. Even though the primiparous cow group had a 16% higher blood glucose concentration while the BHB concentration did not differ, parity did not have an effect on calf health, indicated by no statistical significance in the distribution of calf score groups between cow parity groups (*p* > 0.05).

Cows of the HCS group had more pronounced signs of negative energy balance compared with other groups—higher milk yield, longer rumination time and increased milk fat concentration with increased milk fat to protein ratio—while milk protein and lactose concentrations were lower. The parameters of rumination time, milk yield and milk protein concentration can be used to monitor the metabolic profile of cows, since there were significant differences between all groups. Milk fat, lactose concentrations and milk fat to protein ratio differed between the HCS and LCS groups, but no significant differences were found between the LCS and MCS groups—giving evidence that these parameters should be further investigated. There is good evidence that the metabolic status of the upcoming lactation is predetermined in the dry period and that it has negative effects not only on the health status of the cow, but on the calf as well.

## Figures and Tables

**Table 1:** Comparison of serum glucose concentration in cows and calves according to parity. *n*—number of measurements and animals; *p*—probability.

Indicator	Cow Parity Group	*n*	Mean	Std. Deviation	Std. Error	95% Confidence Interval for Mean		Minimum	Maximum	*p*
						Lower	Upper Bound			
Glucose concentration in cows	Primiparous	20	3.03	0.417	0.093	2835	3225	2.20	3.90	0.004
	Multiparous	20	2.61	0.456	0.102	2396	2824	1.80	3.40	
Glucose concentration in calves	Primiparous	21	6.86	1513	0.33	6173	7551	4.20	11.60	0.605
	Multiparous	21	6.61	1620	0.353	5872	7347	3.70	9.30	

**Table 2:** Comparison of blood serum BHB concentration of cows and calves between parity groups.

BHB	Cow Parity Group	*n*	Mean Rank	Sum of Ranks	*p*
Cows	Primiparous	20	18.65	373	0.311
	Multiparous	20	22.35	447	
Calves	Primiparous	21	21.57	453	0.967
	Multiparous	21	21.43	450	

BHB—β-hydroxybutyrate. *n*—number of animals and measurements. p—probability.

**Table 3:** Distribution of calf score classes among parity groups.

Cow Parity Group	Statistic	Calves’ Score Class		
		Low	Medium	High
Primiparous	*n*	10	9	2
	%	47.6	42.9	9.5
Multiparous	*n*	9	7	5
	%	42.9	33.3	23.8

*X*^2^ = 1.588, df = 2, *p* > 0.05. Low—Low calf score group that scored 5–8 on the health evaluation chart throughout the study period and is considered least susceptible to disease. Medium—Medium calf score group that scored 9–12 on the health evaluation chart throughout the study period and is considered more susceptible to disease. High—High calf score group that scored 14–17 on the health evaluation chart throughout the study period and is considered most susceptible to disease. *n*—number of calves in the calf score group from its respectable cow parity group. Significant results are considered when *p* < 0.05.

**Table 4:** Correlations between cow and calf blood parameters.

		Cow_BHB	Cow_Glu	Calf_BHB	Calf_Glu	Calf Score
Cow_BHB	Correlation Coefficient	-	−0.353 *	−0.476 **	−0.147	−0.05
Cow_Glu	Correlation Coefficient	−0.353 *	-	0.2	0.183	−0.164
Calf_BHB	Correlation Coefficient	−0.476 **	−0.353 *	-	0.096	−0.207
Calf_Glu	Correlation Coefficient	−0.147	0.183	0.096	-	0.111
Calf Score	Correlation Coefficient	−0.05	−0.164	−0.207	0.111	-

* Correlation is significant at the 0.05 level (2-tailed). ** Correlation is significant at the 0.01 level (2-tailed). Cow_BHB—β-hydroxybutyrate concentration of cow blood serum. Cow_Glu—cow blood serum glucose concentration. Calf_BHB—β-hydroxybutyrate concentration of calf blood serum. Calf_Glu—calf blood serum glucose concentration. Calf score—a score assigned according to the calf disease symptom severity.

**Table 5:** Comparison of herd management and milking parameters between parity groups.

	Parity Group	*n*	Mean	Mean Difference	Std. Error	*p*
Rumination time	Primiparous	20	481.21	−32.554	6.086	<0.001
	Multiparous	20	512.77			
Milk yield	Primiparous	20	32.38	−15.016	0.497	<0.001
	Multiparous	20	47.42			
Milk protein concentration	Primiparous	20	3.77	−0.038	0.016	<0.010
	Multiparous	20	3.81			
Milk fat concentration	Primiparous	20	4.24	−0.133	0.032	<0.001
	Multiparous	20	4.38			
Milk fat to protein ratio	Primiparous	20	1.12	−0.025	0.008	<0.001
	Multiparous	20	1.15			
Milk lactose concentration	Primiparous	20	4.61	−0.059	0.004	<0.001
	Multiparous	20	4.67			

*n*—number of animals in group; *p*—probability.

**Table 6:** Comparison of herd management registered parameter means between calf score groups.

Parameter	Groups	*n*	Mean	Std. Deviation	*F*	*p*
Rumination time	Low	19	461.94	134.80	61.86	<0.001
	Medium	16	505.56	108.54		
	High	7	550.79	110.67		
Milk yield	Low	19	38.83	10.70	52.42	<0.001
	Medium	16	36.31	11.62		
	High	7	44.80	14.84		
Milk protein concentration	Low	19	3.84	0.34	29.17	<0.001
	Medium	16	3.78	0.25		
	High	7	3.68	0.36		
Milk fat concentration	Low	19	4.28	0.70	11.45	<0.001
	Medium	16	4.25	0.61		
	High	7	4.47	0.65		
Milk fat to protein ratio	Low	19	1.11	0.16	47.56	<0.001
	Medium	16	1.12	0.14		
	High	7	1.21	0.16		
Milk lactose concentration	Low	19	4.66	0.10	38.76	<0.001
	Medium	16	4.62	0.11		
	High	7	4.61	0.08		

Low—Low calf score group that scored 5–8 on the health evaluation chart throughout the study period and is considered least susceptible to disease. Medium—Medium calf score group that scored 9–12 on the health evaluation chart throughout the study period and is considered more susceptible to disease. High—High calf score group that scored 14–17 on the health evaluation chart throughout the study period and is considered most susceptible to disease. *n*—number of cows. *F*—*F* value. *p*—probability.

**Table 7:** Post hoc analysis of mean differences of herd health management parameters between each calf score group.

Dependent Variable	(I) Calf Score Class	(J) Calf Score Class	Mean Difference (I–J)	Std. Error	*p*
Rumination time	LCS	MCS	−43.62 *	6.46	<0.001
		HCS	−88.85 *	8.28	<0.001
	MCS	LCS	43.62 *	6.46	<0.001
		HCS	−45.22 *	8.40	<0.001
	HCS	LCS	88.85 *	8.28	<0.001
		MCS	45.22 *	8.40	<0.001
Milk yield	LCS	MCS	2.51 *	0.63	<0.001
		HCS	−5.97 *	0.81	<0.001
	MCS	LCS	−2.51 *	0.63	<0.001
		HCS	−8.49 *	0.82	<0.001
	HCS	LCS	5.97 *	0.81	<0.001
		MCS	8.49 *	0.82	<0.001
Milk protein concentration	LCS	MCS	0.07 *	0.01	<0.001
		HCS	0.16 *	0.02	<0.001
	MCS	LCS	−0.06 *	0.01	<0.001
		HCS	0.09 *	0.02	<0.001
	HCS	LCS	−0.16 *	0.02	<0.001
		MCS	−0.09 *	0.02	<0.001
Milk fat concentration	LCS	MCS	0.02	0.03	0.437
		HCS	−0.18 *	0.04	<0.001
	MCS	LCS	−0.02	0.03	0.437
		HCS	−0.21 *	0.04	<0.001
	HCS	LCS	0.18 *	0.04	<0.001
		MCS	0.21 *	0.04	<0.001
Milk fat to protein ratio	LCS	MCS	−0.01	0.01	0.204
		HCS	−0.10 *	0.01	<0.001
	MCS	LCS	0.010	0.01	0.204
		HCS	−0.09 *	0.01	<0.001
	HCS	LCS	0.10 *	0.01	<0.001
		MCS	0.09 *	0.01	<0.001
Milk lactose concentration	LCS	MCS	0.04 *	0.01	<0.001
		HCS	0.05 *	0.01	<0.001
	MCS	LCS	−0.04 *	0.01	<0.001
		HCS	0.01	0.01	0.065
	HCS	LCS	−0.05 *	0.01	<0.001
		MCS	−0.01	0.01	0.065

LCS—Low calf score group. MCS—Medium calf score group. HCS—High calf score group. *—indicates that the difference is significant at the level of *p* < 0.05. *p*—probability.

## Data Availability

The data of the study are publicly available at the link: https://lsmu-my.sharepoint.com/:x:/g/personal/mingurbu0413_kmu_lt/EbB0vdGs451Fg0e8Y9fFfrEBSjGYN0noetVI3fo-9NeVEg?rtime=5BlOKKuN20g (accessed on the 30 July 2023).

## References

[1] Immler M., Büttner K., Gärtner T., Wehrend A., Donat K. (2022). Maternal Impact on Serum Immunoglobulin and Total Protein Concentration in Dairy Calves. Animals.

[2] Schuh K., Häussler S., Sadri H., Prehn C., Lintelmann J., Adamski J., Koch C., Frieten D., Ghaffari M.H., Dusel G. (2022). Blood and Adipose Tissue Steroid Metabolomics and MRNA Expression of Steroidogenic Enzymes in Periparturient Dairy Cows Differing in Body Condition. Sci. Rep..

[3] Belić B., Cincović M., Lakić I., Đoković R., Petrović M., Ježek J., Starič J. (2018). Metabolic Status of Dairy Cows Grouped by Anabolic and Catabolic Indicators of Metabolic Stress in Early Lactation. Acta Sci. Vet..

[4] van Knegsel A.T.M., van den Brand H., Dijkstra J., Tamminga S., Kemp B. (2005). Effect of Dietary Energy Source on Energy Balance, Production, Metabolic Disorders and Reproduction in Lactating Dairy Cattle. Reprod. Nutr. Dev..

[5] Benedet A., Manuelian C.L., Zidi A., Penasa M., De Marchi M. (2019). Invited Review: β-Hydroxybutyrate Concentration in Blood and Milk and Its Associations with Cow Performance. Animal.

[6] Antanaitis R., Juozaitienė V., Malašauskienė D., Televičius M., Urbutis M., Baumgartner W. (2021). Relation of Automated Body Condition Scoring System and Inline Biomarkers (Milk Yield, β-Hydroxybutyrate, Lactate Dehydrogenase and Progesterone in Milk) with Cow’s Pregnancy Success. Sensors.

[7] Klopp R.N., Ferreira C.R., Casey T.M., Boerman J.P. (2022). Relationship of Cow and Calf Circulating Lipidomes with Colostrum Lipid Composition and Metabolic Status of the Cow. J. Dairy Sci..

[8] Jorjong S., van Knegsel A.T.M., Verwaeren J., Lahoz M.V., Bruckmaier R.M., de Baets B., Kemp B., Fievez V. (2014). Milk Fatty Acids as Possible Biomarkers to Early Diagnose Elevated Concentrations of Blood Plasma Nonesterified Fatty Acids in Dairy Cows. J. Dairy Sci..

[9] Argov-Argaman N. (2019). Symposium Review: Milk Fat Globule Size: Practical Implications and Metabolic Regulation. J. Dairy Sci..

[10] Hill T.M., VandeHaar M.J., Sordillo L.M., Catherman D.R., Bateman H.G., Schlotterbeck R.L. (2011). Fatty Acid Intake Alters Growth and Immunity in Milk-Fed Calves. J. Dairy Sci..

[11] Choudhary V., Uaratanawong R., Patel R.R., Patel H., Bao W., Hartney B., Cohen E., Chen X., Zhong Q., Isales C.M. (2019). Phosphatidylglycerol Inhibits Toll-Like Receptor–Mediated Inflammation by Danger-Associated Molecular Patterns. J. Investig. Dermatol..

[12] Stillwell W. (2016). An Introduction to Biological Membranes: Composition, Structure and Function.

[13] Contarini G., Povolo M., Pelizzola V., Monti L., Bruni A., Passolungo L., Abeni F., Degano L. (2014). Bovine Colostrum: Changes in Lipid Constituents in the First 5 Days after Parturition. J. Dairy Sci..

[14] Dewettinck K., Rombaut R., Thienpont N., Le T.T., Messens K., van Camp J. (2008). Nutritional and Technological Aspects of Milk Fat Globule Membrane Material. Int. Dairy J..

[15] Pfrieger F.W. (2003). Cholesterol Homeostasis and Function in Neurons of the Central Nervous System. Cell. Mol. Life Sci..

[16] Sprong R.C., Hulstein M.F.E., van der Meer R. (2002). Bovine Milk Fat Components Inhibit Food-Borne Pathogens. Int. Dairy J..

[17] Zhou X., Xu C., Wang H., Xu W., Zhao Z., Chen M., Jia B., Huang B. (2022). The Early Prediction of Common Disorders in Dairy Cows Monitored by Automatic Systems with Machine Learning Algorithms. Animals.

[18] Angelo I.D.V., Stivanin S.C.B., Vizzotto E.F., Bettencourt A.F., Lopes M.G., Corrêa M.N., Pereira L.G.R., Fischer V. (2022). Feed Intake, Milk Production and Metabolism of Holstein, Gyr and Girolando-F1 Heifers with High Body Condition Score during the Transition Period. Res. Vet. Sci..

[19] Kerwin A.L., Burhans W.S., Mann S., Nydam D.V., Wall S.K., Schoenberg K.M., Perfield K.L., Overton T.R. (2022). Transition Cow Nutrition and Management Strategies of Dairy Herds in the Northeastern United States: Part II—Associations of Metabolic- and Inflammation-Related Analytes with Health, Milk Yield, and Reproduction. J. Dairy Sci..

[20] Overton T.R., McArt J.A.A., Nydam D.V. (2017). A 100-Year Review: Metabolic Health Indicators and Management of Dairy Cattle. J. Dairy Sci..

[21] Cascone G., Licitra F., Stamilla A., Amore S., Dipasquale M., Salonia R., Antoci F., Zecconi A. (2022). Subclinical Ketosis in Dairy Herds: Impact of Early Diagnosis and Treatment. Front. Vet. Sci..

[22] Morales-Piñeyrúa J.T., Damián J.P., Banchero G., Blache D., Sant’Anna A.C. (2022). Metabolic Profile and Productivity of Dairy Holstein Cows Milked by a Pasture-Based Automatic Milking System during Early Lactation: Effects of Cow Temperament and Parity. Res. Vet. Sci..

[23] Televičius M., Antanaitis R., Juozaitienė V., Paulauskas A., Malašauskienė D., Urbutis M., Baumgartner W. (2021). Influence of Calving Ease on In-Line Milk Urea and Relationship with Other Milk Characteristics in Dairy Cows. Agriculture.

[24] NRC (2021). Nutrient Requirements of Dairy Cattle.

[25] Godden S.M., Lombard J.E., Woolums A.R. (2019). Colostrum Management for Dairy Calves. Vet. Clin. N. Am. Food Anim. Pract..

[26] Martin C.C., Baccili C.C., Avila-Campos M.J., Hurley D.J., Gomes V. (2021). Effect of Prophylactic Use of Tulathromycin on Gut Bacterial Populations, Inflammatory Profile and Diarrhea in Newborn Holstein Calves. Res. Vet. Sci..

[27] McGuirk S.M. (2008). Disease Management of Dairy Calves and Heifers. Vet. Clin. N. Am. Food Anim. Pract..

[28] Renaud D.L., Duffield T.F., LeBlanc S.J., Kelton D.F. (2018). Short Communication: Validation of Methods for Practically Evaluating Failed Passive Transfer of Immunity in Calves Arriving at a Veal Facility. J. Dairy Sci..

[29] van Knegsel A.T.M., van den Brand H., Dijkstra J., van Straalen W.M., Jorritsma R., Tamminga S., Kemp B. (2007). Effect of Glucogenic vs. Lipogenic Diets on Energy Balance, Blood Metabolites, and Reproduction in Primiparous and Multiparous Dairy Cows in Early Lactation. J. Dairy Sci..

[30] Santos J.E.P., DePeters E.J., Jardon P.W., Huber J.T. (2001). Effect of Prepartum Dietary Protein Level on Performance of Primigravid and Multiparous Holstein Dairy Cows. J. Dairy Sci..

[31] Wathes D.C., Cheng Z., Bourne N., Taylor V.J., Coffey M.P., Brotherstone S. (2007). Differences between Primiparous and Multiparous Dairy Cows in the Inter-Relationships between Metabolic Traits, Milk Yield and Body Condition Score in the Periparturient Period. Domest. Anim. Endocrinol..

[32] Nichols K., Dijkstra J., Breuer M.J.H., Lemosquet S., Gerrits W.J.J., Bannink A. (2022). Essential Amino Acid Profile of Supplemental Metabolizable Protein Affects Mammary Gland Metabolism and Whole-Body Glucose Kinetics in Dairy Cattle. J. Dairy Sci..

[33] Marumo J.L., Lusseau D., Speakman J.R., Mackie M., Hambly C. (2022). Influence of Environmental Factors and Parity on Milk Yield Dynamics in Barn-Housed Dairy Cattle. J. Dairy Sci..

[34] Sorensen A., Alamer M., Knight C.H. (1998). Physiological Characteristics of High Genetic Merit and Low Genetic Merit Dairy Cows: A Comparison. Proc. Br. Soc. Anim. Sci..

[35] Maekawa M., Beauchemin K.A., Christensen D.A. (2002). Chewing Activity, Saliva Production, and Ruminal PH of Primiparous and Multiparous Lactating Dairy Cows. J. Dairy Sci..

[36] De Boever J.L., Andries J.I., De Brabander D.L., Cottyn B.G., Buysse F.X. (1990). Chewing Activity of Ruminants as a Measure of Physical Structure—A Review of Factors Affecting It. Anim. Feed. Sci. Technol..

[37] Beauchemin K.A., Rode L.M., Yang W.Z. (1997). Effects of Nonstructural Carbohydrates and Source of Cereal Grain in High Concentrate Diets of Dairy Cows. J. Dairy Sci..

[38] Colenbrander V.F., Noller C.H., Grant R.J. (1991). Effect of Fiber Content and Particle Size of Alfalfa Silage on Performance and Chewing Behavior. J. Dairy Sci..

[39] Beauchemin K.A., Rode L.M. (1994). Compressed Baled Alfalfa Hay for Primiparous and Multiparous Dairy Cows. J. Dairy Sci..

[40] Beauchemin K.A., Rode L.M. (1997). Minimum Versus Optimum Concentrations of Fiber in Dairy Cow Diets Based on Barley Silage and Concentrates of Barley or Corn. J. Dairy Sci..

[41] Coulon J.B., Agabriel C., Brunscwig G., Muller C., Bonaiti B. (1994). Effects of Feeding Practices on Milk Fat Concentration for Dairy Cows. J. Dairy Sci..

[42] Gärtner T., Gernand E., Gottschalk J., Donat K. (2019). Relationships between Body Condition, Body Condition Loss, and Serum Metabolites during the Transition Period in Primiparous and Multiparous Cows. J. Dairy Sci..

[43] Dänicke S., Kowalczyk J., Renner L., Pappritz J., Meyer U., Kramer R., Weber E.-M., Döll S., Rehage J., Jahreis G. (2012). Effects of Conjugated Linoleic Acids Fed to Dairy Cows during Early Gestation on Hematological, Immunological, and Metabolic Characteristics of Cows and Their Calves. J. Dairy Sci..

[44] Collazos C., Lopera C., Santos J.E.P., Laporta J. (2017). Effects of the Level and Duration of Maternal Diets with Negative Dietary Cation-Anion Differences Prepartum on Calf Growth, Immunity, and Mineral and Energy Metabolism. J. Dairy Sci..

[45] Leal L.N., Doelman J., Keppler B.R., Steele M.A., Martín-Tereso J. (2021). Preweaning Nutrient Supply Alters Serum Metabolomics Profiles Related to Protein and Energy Metabolism and Hepatic Function in Holstein Heifer Calves. J. Dairy Sci..

[46] Ling T., Hernandez-Jover M., Sordillo L.M., Abuelo A. (2018). Maternal Late-Gestation Metabolic Stress Is Associated with Changes in Immune and Metabolic Responses of Dairy Calves. J. Dairy Sci..

[47] Rossi R.M., Cullens F.M., Bacigalupo P., Sordillo L.M., Abuelo A. (2023). Changes in Biomarkers of Metabolic Stress during Late Gestation of Dairy Cows Associated with Colostrum Volume and Immunoglobulin Content. J. Dairy Sci..

[48] Ha S., Kang S., Jeong M., Han M., Lee J., Chung H., Park J. (2023). Characteristics of Holstein Cows Predisposed to Ketosis during the Post-partum Transition Period. Vet. Med. Sci..

[49] Mee J.F. (2023). Impacts of Dairy Cow Nutrition Precalving on Calf Health. JDS Commun..

[50] De Koster J.D., Opsomer G. (2013). Insulin Resistance in Dairy Cows. Vet. Clin. N. Am. Food Anim. Pract..

[51] Mellado M., Dávila A., Gaytán L., Macías-Cruz U., Avendaño-Reyes L., García E. (2018). Risk Factors for Clinical Ketosis and Association with Milk Production and Reproduction Variables in Dairy Cows in a Hot Environment. Trop. Anim. Health Prod..

[52] Cattaneo L., Piccioli-Cappelli F., Minuti A., Trevisi E. (2023). Metabolic and Physiological Adaptations to First and Second Lactation in Holstein Dairy Cows. J. Dairy Sci..

[53] Duplessis M., Lapierre H., Ouattara B., Bissonnette N., Pellerin D., Laforest J.-P., Girard C.L. (2017). Whole-Body Propionate and Glucose Metabolism of Multiparous Dairy Cows Receiving Folic Acid and Vitamin B12 Supplements. J. Dairy Sci..

[54] Simoni A., Hancock A., Wunderlich C., Klawitter M., Breuer T., König F., Weimar K., Drillich M., Iwersen M. (2023). Association between Rumination Times Detected by an Ear Tag-Based Accelerometer System and Rumen Physiology in Dairy Cows. Animals.

[55] Krpálková L., O’Mahony N., Carvalho A., Campbell S., Walsh J. (2022). Association of Rumination with Milk Yield of Early, Mid and Late Lactation Dairy Cows. Czech J. Anim. Sci..

[56] Toni F., Vincenti L., Grigoletto L., Ricci A., Schukken Y.H. (2011). Early Lactation Ratio of Fat and Protein Percentage in Milk Is Associated with Health, Milk Production, and Survival. J. Dairy Sci..

[57] Pryce J.E., Coffey M.P., Simm G. (2001). The Relationship Between Body Condition Score and Reproductive Performance. J. Dairy Sci..

[58] Bellato A., Tondo A., Dellepiane L., Dondo A., Mannelli A., Bergagna S. (2023). Estimates of Dairy Herd Health Indicators of Mastitis, Ketosis, Inter-Calving Interval, and Fresh Cow Replacement in the Piedmont Region, Italy. Prev. Vet. Med..

[59] Cabezas-Garcia E.H., Gordon A.W., Mulligan F.J., Ferris C.P. (2021). Revisiting the Relationships between Fat-to-Protein Ratio in Milk and Energy Balance in Dairy Cows of Different Parities, and at Different Stages of Lactation. Animals.

[60] Andjelić B., Djoković R., Cincović M., Bogosavljević-Bošković S., Petrović M., Mladenović J., Čukić A. (2022). Relationships between Milk and Blood Biochemical Parameters and Metabolic Status in Dairy Cows during Lactation. Metabolites.

[61] Televičius M., Juozaitiene V., Malašauskienė D., Antanaitis R., Rutkauskas A., Urbutis M., Baumgartner W. (2021). Inline Milk Lactose Concentration as Biomarker of the Health Status and Reproductive Success in Dairy Cows. Agriculture.

[62] Berglund I., Pettersson G., Östensson K., Svennersten-Sjaunja K. (2007). Quarter Milking for Improved Detection of Increased SCC. Reprod. Domest. Anim..

[63] Miglior F., Sewalem A., Jamrozik J., Bohmanova J., Lefebvre D.M., Moore R.K. (2007). Genetic Analysis of Milk Urea Nitrogen and Lactose and Their Relationships with Other Production Traits in Canadian Holstein Cattle. J. Dairy Sci..

[64] Portnoy M., Barbano D.M. (2021). Lactose: Use, Measurement, and Expression of Results. J. Dairy Sci..

[65] Forsbäck L., Lindmark-Månsson H., Andrén A., Åkerstedt M., Andrée L., Svennersten-Sjaunja K. (2010). Day-to-Day Variation in Milk Yield and Milk Composition at the Udder-Quarter Level. J. Dairy Sci..

[66] Noya A., Casasús I., Ferrer J., Sanz A. (2019). Long-Term Effects of Maternal Subnutrition in Early Pregnancy on Cow-Calf Performance, Immunological and Physiological Profiles during the Next Lactation. Animals.

[67] Osorio J.S., Trevisi E., Ballou M.A., Bertoni G., Drackley J.K., Loor J.J. (2013). Effect of the Level of Maternal Energy Intake Prepartum on Immunometabolic Markers, Polymorphonuclear Leukocyte Function, and Neutrophil Gene Network Expression in Neonatal Holstein Heifer Calves. J. Dairy Sci..

